# Impact of Phonon Surface Scattering on Thermal Energy Distribution of Si and SiGe Nanowires

**DOI:** 10.1038/srep25818

**Published:** 2016-05-13

**Authors:** Abhinav Malhotra, Martin Maldovan

**Affiliations:** 1 School of Chemical & Biomolecular Engineering, Georgia Institute of Technology, Atlanta, Georgia 30332, USA; 2 School of Physics, Georgia Institute of Technology, Atlanta, Georgia 30332, USA

## Abstract

Thermal transport in nanostructures has attracted considerable attention in the last decade but the precise effects of surfaces on heat conduction have remained unclear due to a limited accuracy in the treatment of phonon surface scattering phenomena. Here, we investigate the impact of phonon-surface scattering on the distribution of thermal energy across phonon wavelengths and mean free paths in Si and SiGe nanowires. We present a rigorous and accurate description of phonon scattering at surfaces and predict and analyse nanowire heat spectra for different diameters and surface conditions. We show that the decrease in the diameter and increased roughness and correlation lengths makes the heat phonon spectra significantly shift towards short wavelengths and mean free paths. We also investigate the emergence of phonon confinement effects for small diameter nanowires and different surface scattering properties. Computed results for bulk materials show excellent agreement with recent experimentally-based approaches that reconstruct the mean-free-path heat spectra. Our phonon surface scattering model allows for an accurate theoretical extraction of heat spectra in nanowires and contributes to elucidate the development of critical phonon transport modes such as phonon confinement and coherent interference effects.

Understanding and controlling nanoscale heat transport phenomena can significantly benefit many research areas, including thermoelectric devices, micro and nano-electronics, heat assisted magnetic recording, and nano-manufacturing^1^. For example, the performance of thermoelectric materials is determined by the figure-of-merit *ZT* = *S*^*2*^*σ/κ*, which requires a low thermal conductivity *κ* and a high electrical conductivity *σ* to obtain enhanced energy-conversion efficiencies. The prospects of independent manipulation of thermal and electrical transport properties have made semiconductors a material of choice for thermoelectrics. In particular, a large number of outstanding low-thermal conductivity highly-efficient thermoelectric materials have been recently introduced, including nanowires^2^,^3^,^4^,^5^,^6^,^7^,^8^,^9^,^10^,^11^,^12^,^13^,^14^,^15^,^16^,^17^,^18^, nano-membranes/thin-films^19^,^20^,^21^,^22^,^23^, nanomeshes^24^,^25^,^26^,^27^,^28^ and polycrystals^29^,^30^,^31^.

A current fundamental challenge in nanoscale heat transport is to precisely predict how much heat is carried by phonons with different wavelengths and mean free paths. In recent years, significant efforts have been conducted to reconstruct the mean-free-path accumulative thermal conductivity of bulk materials via the combination of the suppression function and experimental measurements^32^,^33^,^34^,^35^. This recently proposed experimental route to obtain heat spectra can be readily compared to DFT-based numerical calculations^36^. Importantly, if such deep knowledge of fundamental phonon transport properties in bulk materials is to be routinely extended to nanostructures, an accurate description of phonon surface scattering is critically needed beyond the standard assumption of complete diffuse scattering. In particular, a major research question that needs to be answered is the precise determination of the proportion of thermal phonons that are specularly and diffusively scattered at surfaces. Historically, the boundary scattering description has relied on an empirical approach (i.e. the Matthiessen’s rule) to find an overall relaxation time that accounts for both internal and boundary scattering processes. There is an inherent inaccuracy with the use of Matthiessen’s rule, as it assumes phonon boundary scattering as an internal scattering process and does not capture in detail the actual physical scattering mechanisms occurring at the boundaries. In addition, in the literature, the effects of phonon diffusive scattering from the boundaries have been accounted for by assuming a constant value for the surface specularity *p* (i.e. the proportion of specularly reflected phonons). This formulation, however, does not distinguish among the scatterings for phonons with different wavelengths. Another expression for the specularity *p* is the formula obtained by Ziman^37^ (there are several instances of incorrect usage of this formula with an extra π in the exponent in literature). Ziman’s formula is an improvement over the constant *p* model as it accounts for momentum dependent scattering but its weakness lies in failing to account for incident angle dependence of phonon surface scattering. As a result, there is currently a need for accurately describing phonon-surface interactions in order to understand, predict, and control heat conduction processes at the nanoscale. Without achieving such fundamental understanding it will be difficult to precisely establish the development of confinement, ballistic, and coherent interference effects on heat conduction and to be able to rationally design nanostructures for thermal applications.

Here, we present a rigorous and accurate approach for thermal phonon heat conduction by introducing the Beckmann-Kirchhoff based surface scattering theory for thermal phonons and the effects of surface shadowing to account for real surface behaviour. We use our theoretical approach to provide a quantitative analysis on the distribution of thermal energy among different phonon wavelengths and mean free paths in Si and SiGe nanowires. Specifically, we show how the phonon heat spectra is significantly modified by the nanowire surface conditions. Decreased diameters and increased surface roughness and correlation lengths create the largest shifts towards short phonon wavelengths and mean free paths (analogous to blue-shift in optics) while alloying defects generate an opposite effect (red-shift). We also employ our wavelength heat spectrum calculations to predict and study the emergence of phonon confinement effects in Si and SiGe nanowires at different diameters and surface conditions. The results of this paper provide a critical step forward towards application-specific rational design of thermal materials through precise manipulation of phonon transport properties.

## Theory

**Figure 1:**
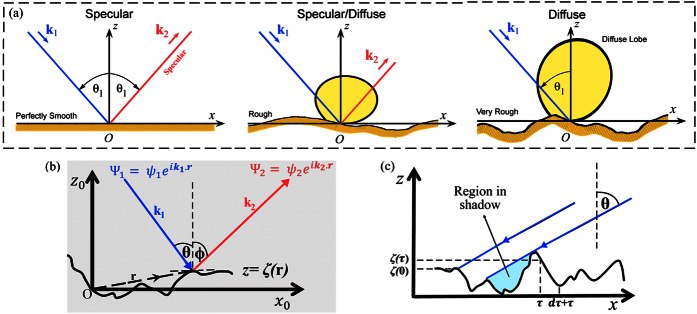
Schematics of phonon surface scattering. (**a**) Transition from specular reflection to diffuse scattering. *Left*: Perfectly smooth surface showing perfect reflection. *Centre*: Rough surface with partially specular/partially diffuse scattering. *Right*: Very rough surface exhibiting complete diffuse scattering. (**b**) Incident Ψ_1_ and scattered Ψ_2_ phonon fields with angle of incidence θ and scattered angle ϕ from a rough surface *z* = ζ(r). **r** is the position vector and **k**_1_ and **k**_2_ are the corresponding wavevectors. (**c**) Surface shadowing. Phonons are incident with wavevector **k** at angle of incidence θ measured clockwise from the z axis. Shadows are cast along the -x direction reducing the surface area available for scattering.

The accurate description of boundary scattering effects is critically needed to understand and control nanoscale heat transport. In nanostructures, the presence of surfaces provides additional scattering mechanisms for phonons to attain local equilibrium. Intuitively, one would expect the specific surface features to influence the mechanisms for phonon scattering and heat transport. Two most common variables to characterize a surface are the variation from its mean plane (i.e. roughness) and the distance between two roughness features (i.e. correlation length). From a practical standpoint, the effects of a surface can only be described statistically since a point-to-point description would be nearly impossible. Here, we employ a rigorous surface scattering theory − the quasi-classical Beckmann-Kirchhoff (BK) approach^38^, which has been extensively used in other fields such as electromagnetics and acoustics but its application to heat transport has been largely unexplored. A completely statistical analysis of surface scattering properties yields the Beckmann-Kirchhoff surface scattering model, where the distribution of the scattered thermal energy [Fig. 1] is predicted by an analytical formula giving the specific dependence on incident and reflected angles, wavevectors, surface roughness and correlation lengths (See Methods). In particular, *the proportion of specularly scattered phonons* from the surface is given by *p* = exp(−4*η*^2^*k*^2^cos^2^*θ*). It is important to note that the Beckmann-Kirchhoff formulation relies on certain central assumptions: (1) the surface is large as compared to the wavelength. (2) The boundary behaves as a perfectly free surface. (3) The field on the local tangent plane can approximate the field at any point on the surface (also referred to as the Kirchhoff’s approximation or tangent-plane method). (4) The effects at the edges of the surface and shadowing are neglected. (5) Only a single scattering event from the surface roughness is considered. We analyse the general validity of these assumptions for thermal transport in silicon nanowires. Typical nanowire surface lengths are in the scale of few microns which are larger than the dominant heat-carrying phonon wavelengths on the nanometre scale^9^,^39^. In addition, there is an expected decrease in the heat-carrying wavelengths in nanowires due to boundary scattering, which is detailed below in the heat spectrum discussion. The large mechanical contrast for solid-air interfaces leads to a very large impedance ratio, rendering the free surface approximation essentially true. The validity of tangent plane approximation requires a surface correlation length of the order of the incident phonon wavelength. An analysis of surface correlation lengths found in experiments and heat-carrying phonon wavelengths reveals the validity of this approximation in typical Si nanowires (see Supplementary Table S1 and Fig. S2). Here, we broaden the BK model for application to heat transport by including rough surface shadowing. Shadowing is the physical phenomena where a portion of the surface is hidden from the incident wave by other points on the surface [Fig. 1c]. It is well understood^40^,^41^ that shadowing plays an important role in accurately predicting the scattered intensity, particularly at grazing incident angles. To account for this phenomena mathematically, a shadowing function defined as the ratio of the unscreened surface to the total surface is introduced, which takes the value unity for points not in shadow and zero for shaded parts of the surface. We thus extend the Beckmann-Kirchhoff scattering model by multiplying the surface area with the spatially averaged shadowing function.

In conjunction with the previous rigorous surface scattering approach, the effects of the boundaries are taken into account by calculating the reduced phonon mean free paths *ℓ*_**k**_. Unlike the Matthiessen’s rule, we use the fundamental definition of the phonon mean free path (see Supplementary Information) and our approach therefore distinguishes between the discrete nature of boundary scattering (i.e. phonon-surface scattering) and the volumetric nature of internal scattering events (e.g. phonon-phonon, phonon-impurity scattering). The accurate establishment of the bulk phonon relaxation time *τ*_0_ is currently a very active research area involving experimental techniques and theoretical models ranging from first-principle to phenomenological approaches^32^,^33^,^34^,^35^,^36^. Overall, the different approaches yield the same range of relaxation times for silicon (see Supplementary Fig. S3). In our work, we use expressions^15^ having a *ω*^−2^ and *ω*^−4^ dependence of relaxation times by Umklapp scattering and isotope scattering effects, respectively. These dependences have been demonstrated originally by Slack^42^ and Klemens^43^. Optical modes are neglected, as owing to their low group velocities the contribution to thermal conductivity is low^21^. For calculation of phonon transport properties and thermal conductivities, we use the linear Boltzmann transport equation under the relaxation time approximation. The thermal conductivity *κ* is given by the well-known formula 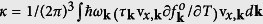, where *ħ* is the Planck constant, *ω*_**k**_ is the phonon frequency, *f*_**k**_^o^ is the equilibrium distribution function, and the product of the relaxation time *τ*_**k**_and the velocity *v*_**k**_ is the *reduced* mean free path *ℓ*_**k**_.

## Discussion

**Figure 2:**
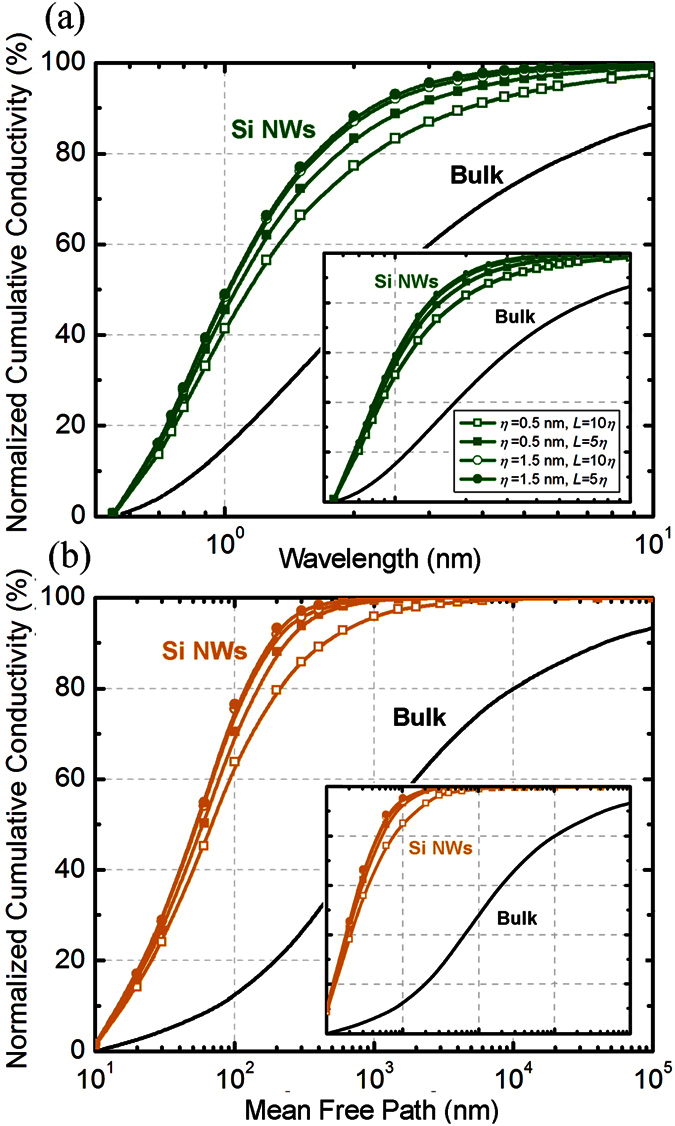
Numerical prediction for heat spectra of silicon nanowires at room temperature. (**a**) Wavelength spectrum and (**b**) Mean-free-path spectrum for diameter *d* = 100 nm. Inset shows the calculations for *d* = 30 nm. Heat spectra are plotted for different roughness *η* and correlations lengths *L*. Bulk silicon spectra is also shown as reference.

Our first goal is to elucidate the thermal energy distribution among phonons with different wavelengths and mean free paths in Si and SiGe nanowires under different boundary conditions. Using our surface scattering model, we calculate the wavelength and mean-free-path heat spectra for Si nanowires for different diameters, surface roughness, and correlation lengths. Figure 2 quantitatively shows how the introduction of rough boundaries strongly shifts the nanowire heat spectrum to shorter wavelengths and shorter mean free paths with respect to bulk silicon. For example, in bulk Si, the dominant heat carrying phonons (10–90%) have a wavelength λ ~ 0.9–10 nm while in a nanowire of 100 nm diameter [Fig. 2a], the dominant spectrum shifts to 0.6–2.5 nm range. A similar effect is seen in the mean-free-path spectrum which shows a marked shift from 0.1 μm-100 μm (bulk) to 15–200 nm (nanowire) [Fig. 2b]. Such a spectrum shift is a direct consequence of the transition from bulk to a nanowire structure. In general, with the introduction of the boundaries, phonons with longer mean free paths (and larger wavelengths) can interact more strongly with the boundaries than those with shorter mean free paths. Since the roughened boundary provides an additional scattering mechanism, these phonons scatter more and achieve local equilibrium. The additional boundary scattering not only reduces the nanowire thermal conductivity as compared to bulk, but also strongly modifies the phonon heat spectra. Figure 2 also shows the marked shift to shorter wavelengths and mean free paths with increasing surface roughness. By increasing the roughness from 0.5 nm to 1.5 nm, there is a shift to shorter wavelengths and smaller mean-free-paths in the thermal phonon distribution caused by the enhanced diffuse scattering. We also investigated the effects of the surface correlation lengths on the heat spectra. For a given surface roughness, a smaller correlation length enhances the impact of scattering at the boundary. This is seen as an enhanced shift in the heat spectra towards shorter mean free paths and smaller wavelengths (i.e. blue-shift). For example, in a Si nanowire with *d* = 100 nm and surface roughness *η* = 0.5 nm, phonons with wavelengths shorter than 2 nm carry ~77% of total heat if the correlation length is *L* = 5 nm. However, this proportion increases to >83% if the correlation length is shortened to *L* = 2.5 nm. Similarly, in the case of the mean-free-path spectrum, phonons with mean-free-paths less than 200 nm carry 79% of heat if *L* = 5 nm. Reducing this correlation length to *L* = 2.5 nm increases the heat carried by these phonons to 88%. Importantly, these detailed modifications of the heat spectra cannot be predicted by approximate boundary scattering models. We note that the surface correlation length provides a statistical quantification of the distance between repeated roughness features. The frequent appearance of “hills and valleys” for a surface with small correlation length contributes towards more shadowing, modifying the probability of specular reflection. The enhanced shadowing for small correlations lengths increases the overall boundary effect causing the nanowire heat spectra to be dominated by shorter wavelength and smaller mean-free-paths. We also performed a comparison of the previous results with the spectrums for a smaller nanowire (*d* = 30 nm) and found an additional shift of the spectrum to lower wavelength and mean free paths (Fig. 2, insets). This is consistent since the effects of boundary scattering are slightly more pronounced in a smaller diameter nanowire because a larger range of phonons can interact strongly with the nanowire surfaces.

**Figure 3:**
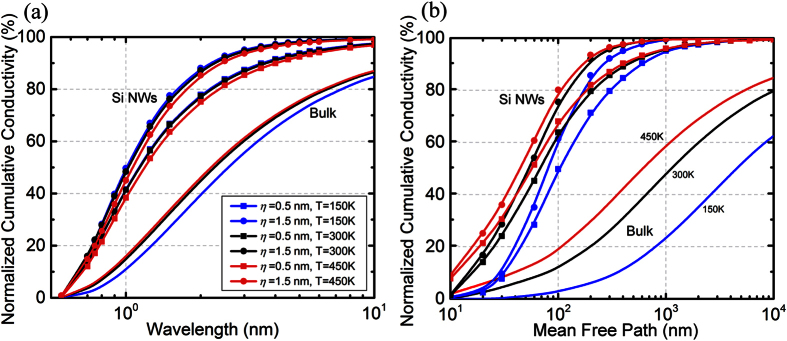
Temperature dependence of heat spectra for Si nanowires with diameter *d* = 100 nm and correlation lengths *L* = 10*η* as a function of (**a**) Wavelength and (**b**) Mean-free-path, calculated at temperatures *T* = 150 K, 300 K, and *T* = 450 K (blue, black and red colours respectively). Surface roughness values are *η* = 0.5 nm and 1.5 nm

Because heat conduction is in general significantly dependent on temperature, we investigate the effect of temperature on the nanowire heat spectrums. Figure 3 shows that a temperature change from 150 K to 450 K modifies the heat spectrums for the same surface conditions. This can be explained by considering the fundamental scattering processes involved – phonon-phonon scattering, isotope scattering and boundary scattering processes. The first two are internal scattering mechanisms since they occur throughout the volume of the nanowire. The change in temperature influences the phonon-phonon scattering processes and modifies the phonon mean free paths. The boundary scattering process itself does not depend on temperature (i.e. the specularity of the surface depends on incoming phonon wavevector and surface conditions). Thus, a temperature dependent heat spectrum in the case of Silicon nanowires is an indicator for quasi-ballistic transport, where both internal and boundary scattering of phonons influence heat transfer.

**Figure 4:**
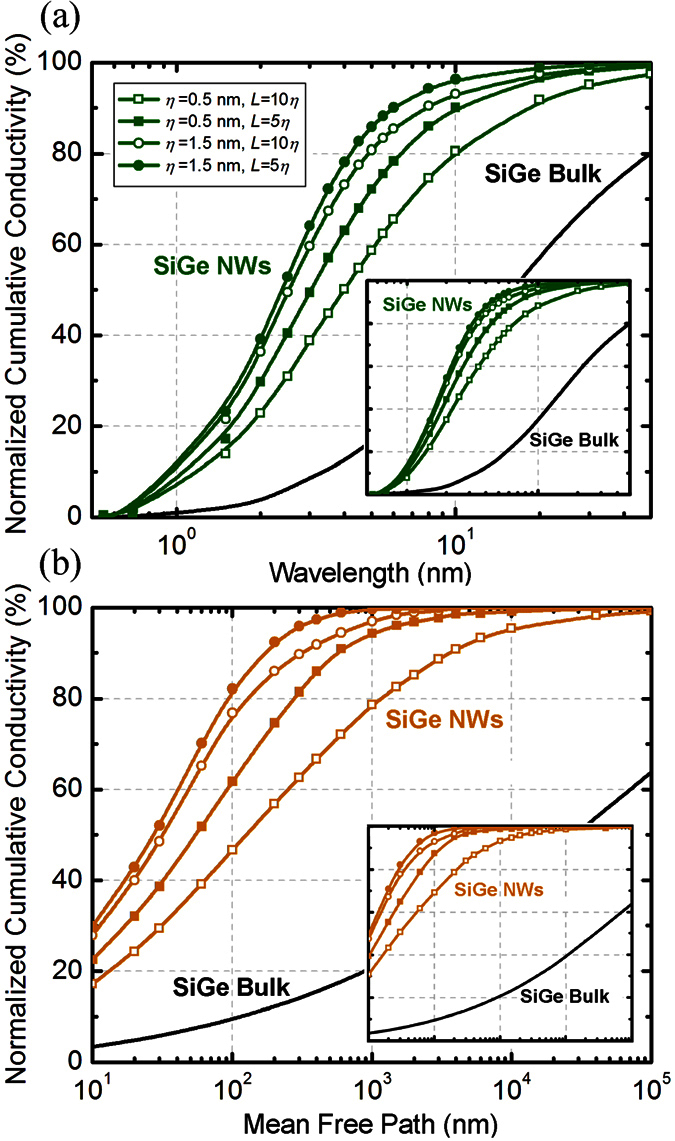
Wavelength heat spectra for Si_0.9_Ge_0.1_ alloy nanowires at room temperature for (**a**) *d* = 100 nm (and inset for *d* = 30 nm). (**b**) Mean-free-path heat spectrum for nanowire diameters *d* = 100 nm and 30 nm.

**Figure 5:**
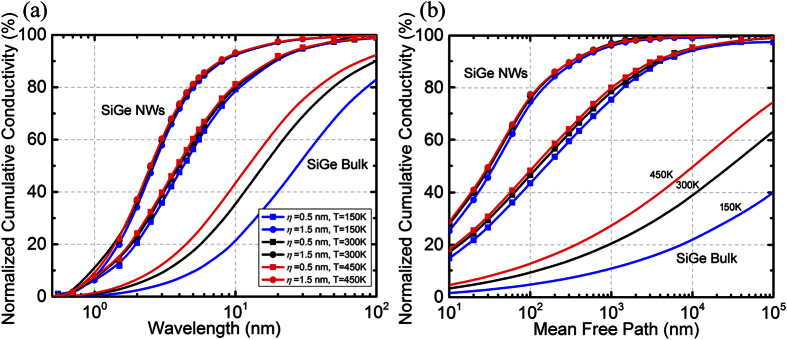
The dependence of (**a**) Wavelength and (**b**) Mean-free-path heat spectra on temperature for Si_0.9_Ge_0.1_ nanowires with diameter *d* = 100 nm, correlation length *L* = 10*η*, and surface roughnesses *η* = 0.5 nm and 1.5 nm calculated at temperatures *T* = 150 K, 300 K, and *T* = 450 K are shown by blue, black and red lines respectively.

It is important to note that, in contrast to surface scattering, the addition of mass-defects in the Si crystalline lattice in the form of Ge atoms is an effective mechanism to reduce the thermal conductivity by scattering phonons with shorter wavelengths and smaller mean free paths^27^,^44^. For this reason, the dominant wavelengths and the mean free paths in low Ge concentration SiGe alloys are higher as compared to pure silicon. This means that while surface scattering shifts the bulk heat spectra to short wavelengths, alloy scattering shifts the bulk spectra to long wavelengths. We found that in a SiGe nanowire, where the spectra are already shifted in comparison with Si, the effect of boundary scattering is again seen by a transition to shorter mean free paths and wavelengths (i.e. blue-shift) [Fig. 4]. This is similarly explained by the ability of the phonons with longer wavelengths (and mean free paths) to “see” the boundaries more effectively and have a higher interfacial interaction. An interesting property of the alloyed nanowire spectrum is the larger and broader ranges of the dominant region. If we consider the range of the middle 80% of the heat spectrum (10–90% of heat), the phonon mean free paths lie in the range of 5 nm-3μm while the wavelengths are in the range of 1–20 nm. The exact value certainly depends on the roughness and correlation length of the surface under consideration. This is significantly larger and wider than the ranges seen for Si nanowires. These spectrum features are a consequence of the combined effects of alloyed scattering and boundary scattering. We note that while Ge atoms are very effective in scattering short mean free path (and small wavelength) phonons, boundaries are effective in interacting with phonons of larger mean free paths (and longer wavelengths). This leaves a larger and relatively broader “middle” range as the major conductor of the heat in the SiGe alloyed nanowires. The effects of reducing the correlation lengths via the enhanced shadowing of nanowire surface is still observed similarly to Si nanowires. Lastly, as seen in Fig. 5, a weak temperature dependence of the heat spectra is observed in the case of alloyed nanowires. Note that the thermal transport in these nanowires is predominantly *ballistic* due to the fact that the nanowire diameters (*d* ~ 30 − 100 nm) are much smaller compared to the dominant phonon mean free paths. This causes the boundary scattering to be the primary relaxation mechanism (i.e. Casimir regime^37^) leading to heat spectra that are almost independent of temperature.

**Figure 6:**
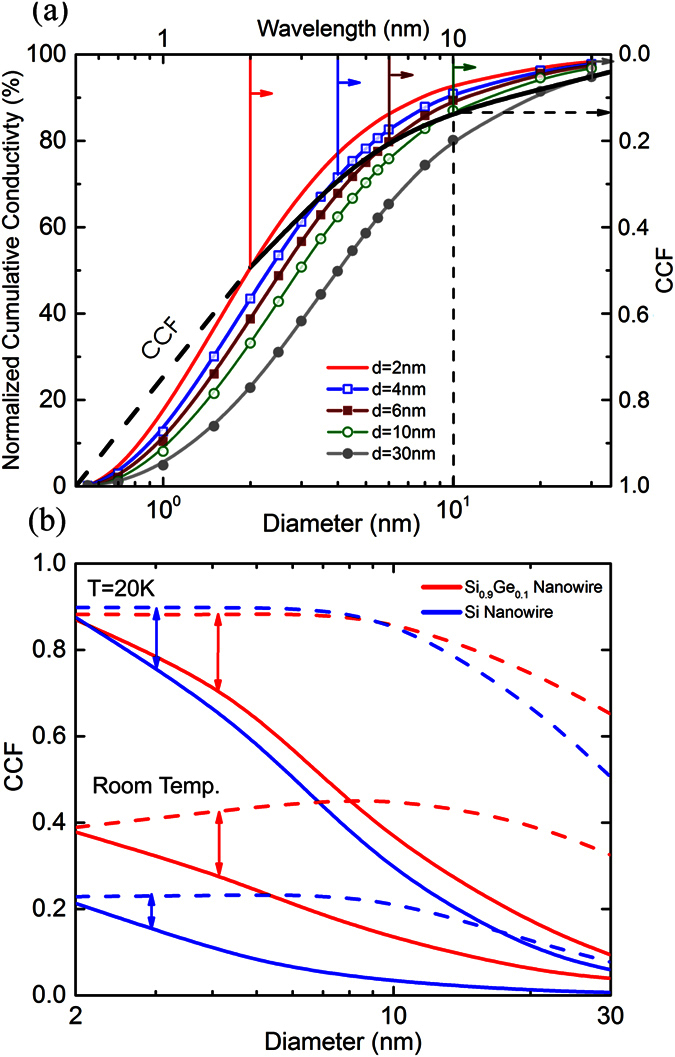
(**a**) Calculation of Confinement Contribution Fraction (CCF) for Si_0.9_Ge_0.1_ alloy at room temperature from the wavelength heat spectrum for *d* = 2, 4, 6, 10 and 30 nm. CCF is significant for nanowire dimensions approaching Si lattice constant. (**b**) CCF values for Si (blue) and Si_0.9_Ge_0.1_ (red) nanowires as a function of diameter for room temperature and *T* = 20 K. CCF curves provide the minimum (solid) and upper range (dashed) to possible contribution of confinement wave effects in a particular nanowire. An increasing CCF is an indicator of enhanced quantum confinement for smaller diameters.

The accurate prediction of phonon heat spectra in nanostructured materials under different surface conditions can be used to establish important heat transport regimes such as phonon confinement or coherent interference. For instance, we next employ our calculation of the wavelength heat spectrum in nanowires to study the emergence of phonon confinement effects. As the diameter *d* is reduced, phonon confinement can develop as a result of the phonon reflections at the boundaries. For these effects to effectively occur in the nanowire, the phonon wavelengths need to be at least of the order of the diameter^45^. Additionally, such quantum confinement effect is expected when the phonons maintain the phase after surface scattering. We consider a nanowire of small surface roughness (0.5× lattice constant) and correlation length (10*η*) (see supplementary information for additional surface conditions). To quantify the possibility of the occurrence of the confinement effects, we define a Confinement Contribution Fraction (CCF) generated from the phonon energy distribution curves as shown in Fig. 6. CCF curves provide the minimum (λ ≥ 2*d*′) and upper range (λ > 2*d*′/5) to possible contribution of confinement wave effects in a particular nanowire (*d*′ = *d*
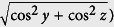
. For example, CCF_min_ = 0.13 is obtained for Si_0.9_Ge_0.1_ alloyed nanowires at room temperature with *d* = 10 nm. We note that confinement is expected to play an increasing role for smaller diameters as indicated by larger CCF values. We predict that for *d* = 2 nm confinement is possible for nearly half (CCF ~ 0.40) of the heat-carrying phonons. At reduced temperature of 20 K, higher CCF values indicate a larger proportion of phonons satisfying the confinement conditions and predicts the possibility of manipulating heat using coherent scattering. Unlike alloyed nanowires, for Si nanowires at room temperature confinement effects are much smaller for *d* > 10 nm. However, we show that at very small diameters (*d* = 2 nm) significant confinement may be seen with a maximum contribution of 23% for heat-carrying phonons. At low temperatures, the confinement effects are stronger due to an overall reduction in phonon-phonon scattering.

**Figure 7:**
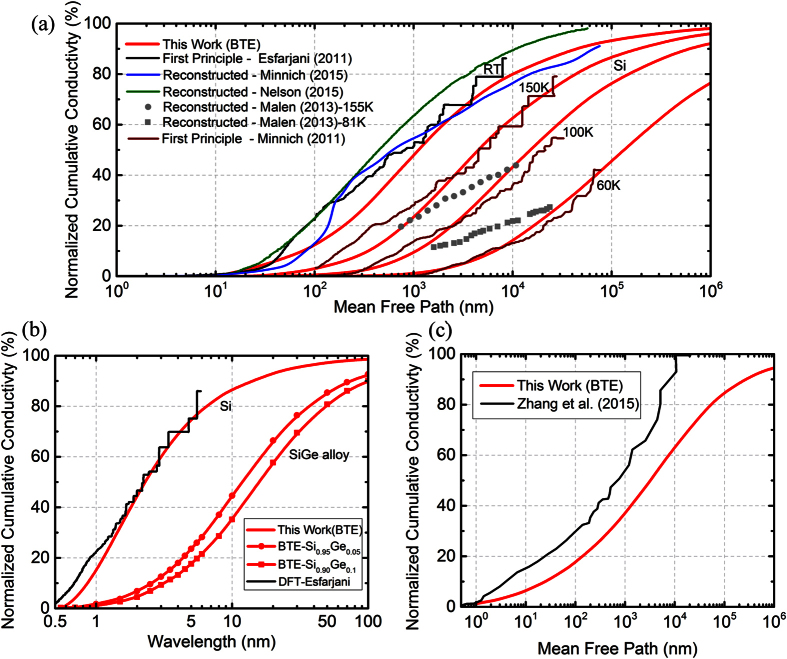
(**a**) Comparisons between mean-free-path heat spectrum (normalized cumulative conductivity) for bulk Si from our calculations, first principle approaches, and reconstruction from experiments^32^,^33^,^34^,^35^,^36^ (**b**) Calculated wavelength heat spectrum for bulk Si and SiGe alloy, and (**c**) Calculated mean-free-path heat spectrum for Si_0.9_Ge_0.1_ nanowire and the reconstructed spectrum using Ziman’s formula for *η* = 0.1 nm^35^.

In addition to Si and SiGe nanowires, we also predict the heat spectrum of *bulk* Si and SiGe materials and compare it against computationally expensive DFT calculations^36^ and more recent experimental approaches that reconstruct the conductivity measurement into a heat spectrum as a function of phonon mean free path by use of a suppression function^32^,^33^,^34^,^35^. We note the strong agreement between all approaches in Fig. 7a enabling a prediction about the bulk silicon mean-free-path heat spectrum with certainty. Another important feature that needs to be considered is the thermal phonon wavelengths. Since the experimental reconstruction approach for the heat spectrum can provide only the mean free path spectra, we are restricted to compare our bulk calculations to DFT in Fig. 7b. Once again our calculations for bulk silicon are in close agreement with DFT calculations^36^. In addition, we present our predictions for the heat spectrum of bulk Si_0.9_Ge_0.1_ alloy. As mentioned previously, with the introduction of Ge atoms in the matrix, the spectrum shows a marked change towards longer phonon wavelengths (and larger mean free paths). It can thus be postulated that any change from a pure bulk structure (e.g. defects, interfaces etc.) affects both the thermal conductivity and the phonon heat spectrum. For the case of nanowires, the reconstructed heat spectrum as a function of mean free paths is available only for a Si_0.9_Ge_0.1_ nanowire^35^. This reconstruction is based on a Ziman formulation (normal incidence) for phonon boundary scattering which is expected to overestimate the effect of the boundaries (see Supplementary Fig. S5). Our results using the BTE formulation with the same assumptions show a close agreement with this reconstructed mean free path in Fig. 7c. From the perspective of material design and heat manipulation applications, the knowledge of heat spectrum is essential and thus needs to be accurately determined by using surface scattering models that account for all relevant physical variables.

**Figure 8:**
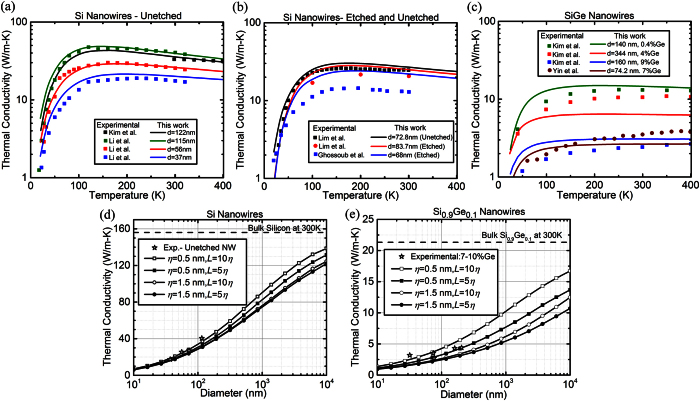
Calculations from our model for (**a**) Unetched Si nanowires, (**b**) Unetched and etched Si nanowires and (**c**) SiGe nanowires for different diameters *d*. Comparison of the thermal conductivity as a function of temperature from our model shows agreement with experimental measurements (symbols)^4^,^39^,^46^,^49^,^51^. (**d**,**e**) Theoretical predictions using our approach (BK method with shadowing) for nanowires of different diameters at room temperature with different surface characteristics (roughness and correlation length) for pure Si and SiGe alloys, respectively.

We additionally use our surface scattering model (i.e. Beckmann-Kirchhoff formulation with shadowing and angle and momentum dependent phonon scattering) to predict Si and SiGe nanowire thermal conductivities. We find excellent agreement with experimental dataset for the unetched nanowires of diameters 115 nm, 56 nm, 37 nm^4^ and 122 nm^46^ in Fig. 8. Since no surface parameters were reported in these experiments, we assume a boundary disorder in the order of Si crystal unit cell (0.4 nm-0.6 nm) and a large correlation (10*η*) as the wires were not roughened intentionally. Our accurate predictions in unetched nanowires can be further seen in Fig. 8b where using the previous assumptions about surface parameters, we also find a good comparison with the experimental values^39^. For etched nanowires, the theoretical calculations are in agreement with experiments^39^,^47^ but the results are slightly larger than the experimental values. The inclusion of an amorphous layer^28^,^48^ at the boundary (to account for the chemically etched surfaces) is insufficient to account for this reduction. This enhanced reduction could be the result of microstructural changes which appear as a result of the etching process, as suggested by D. Cahill^49^. Additional strains in the silicon crystal introduced due to these changes were not included in our model. We also apply our model to SiGe alloy nanowires and found a similar agreement in Fig. 8c with the experimental data by taking a higher value of roughness (~3× Si lattice constant). This is consistent with a previous comparison^50^ which uses a boundary scattering description with *p* = 0. We note that accounting for changes in relaxation times due to mass difference of point defects^27^,^44^,^50^ and experimental measurements^46^,^51^ are very sensitive to impurity (alloy) fraction, a slight inaccuracy or mismatch in the reported defect percent against the actual fraction could account for the observed lowered conductivity. Interestingly, for both Si and SiGe nanowires, the BK model provides agreement with experimental results even at low temperatures where the minimum correlation length for strict applicability of the Kirchhoff approximation is expected to be higher. In addition, we predict the thermal conductivity of Si and SiGe nanowires as a function of diameter for different roughness and correlation lengths. The numerical predictions for Si nanowires in Fig. 8 show how the diameter reduction lowers the thermal conductivity due to the emergence of the non-diffusive (quasi-ballistic) regime of heat conduction where phonon-interface scattering plays an important role. The thermal conductivity is higher for higher correlation lengths as the probability of phonons undergoing specular reflection is higher due to the larger distance between two surface features. We also predict the thermal conductivities for alloyed nanowires and a diameter dependence for Si_0.9_Ge_0.1_ nanowires shows a similar trend as seen in Si for a quasi-ballistic regime. The magnitude of thermal conductivities in this case, however, are one order of magnitude smaller due to alloying effects. Our model with all the relevant physical parameters and surface scattering phenomena is thus able to predict the thermal conductivities of Si and SiGe nanowires. The presence of crystal strains and/or additional impurities in the samples could lead to lower thermal conductivities (as observed experimentally for etched nanowires).

## Summary

In summary, we have developed an accurate approach to study heat transport at the nanoscale by following a systematic treatment of phonon surface scattering effects. We utilized the Beckmann-Kirchhoff surface scattering theory to accurately model the phonon-interface interactions as well as introducing the physical phenomena of rough surface shadowing. All pertinent physical variables (phonon momentum, angle of incidence at the surface, surface roughness and correlation lengths) are therefore included in our approach. We predicted the modified wavelength and mean-free-path heat spectra in Si and SiGe nanowires for different diameters (*d* = 100, 30 nm) and structural surface conditions (*η* = 0.5, 1.5 nm, *L* = 10*η*, 5*η*). The dominant phonon wavelengths in bulk Si are λ ~ 1–10 nm but are modified to be <2 nm for a Si nanowire of 100 nm diameter. Bulk dominant mean-free-paths of 0.1 μm-100 μm are shortened to 20 nm-200 nm in these nanowires. The use of SiGe alloyed nanowires allows to increase the wavelength and mean free paths in the heat spectra. The dominant wavelength of phonons and mean free paths carrying the majority of heat in these nanowires is of the range λ ~ 1–20 nm and 5 nm-3 μm, respectively. We also find that boundary scattering is the dominant scattering process at this length scale for alloyed nanowires. We also studied and quantified phonon confinement in Si and SiGe nanowires. SiGe nanowires are expected to show significant confinement effects (~90% at 20 K and *d* = 2 nm) under smooth surface conditions and small nanowire sizes, especially at low temperatures. The effect of confinement for Si nanowires is predicted to be small for *d* > 10 nm at room temperature. However, significant quantum effects for Si at small diameters and low temperatures can be observed. The accuracy of our approach is seen by comparison with experimental measurements and mean free path spectra. The improved description of phonon surface scattering and prediction of heat spectra would allow for the precise design of nano-engineered devices as well as the fundamental understanding on nano-scale heat transport in order to obtain emerging novel thermal effects such as phonon coherent interference.

## Methods

### Surface Scattering Model

Considering a random 1-D rough surface, where any point can be represented by its position vector [Fig. 1b]


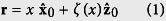


By dropping the factor exp(−*iωt*) for sake of brevity, the incident field can be written as


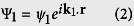


where *ψ*_1_ is the amplitude of the incident wave. Writing the Helmholtz wave equation for the total field ψ


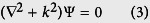


where *k* denoted the modulus of the wavevector **k**, Green’s function theory gives the solution of the wave equation at an interior point in terms of the values of the function ψ and its normal derivative on the surface *S*. The scattered field Ψ_2_ at a point of observation *X* located at a distance *d* from a point (*x, ζ*(*x*)) at the surface is given by


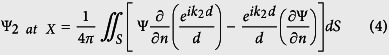


To obtain the wave function and its derivative at the surface, the Kirchhoff approximation is utilized. This assumption approximates the field at the surface to be the same as the field at a local tangent plane at the point (*x, ζ*(*x*)). Physically, the validity of this approach hinges on the fact that there should be no sharp points on the surface. Under this assumption, we can write^45^:


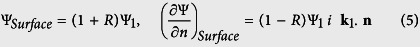


where *R* denotes the reflection coefficient for a smooth plane and **n** is the normal to the surface. In general, *R* may depend on the angle of incidence of the field and the physical properties of the media at both sides of the surface. However, we consider a perfectly free surface (due to the large mechanical contrast for solid-air interfaces) and therefore *R* = −1. Under the above formulation, the normalized scattered field from a one-dimensional surface yields the following expression^38^


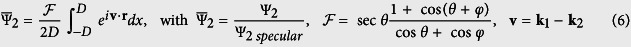


where the surface extends from −*D* to *D*. 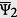 is the ratio of the scattered field to the scattered field in the specular direction by a smooth, perfectly free surface with the same dimensions. A statistical description of a rough surface requires the knowledge of two basic parameters; firstly, the variation in height and secondly, the correlation between these surface irregularities. We consider a one-dimensional rough surface which varies in height *ζ* along the direction 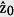 and extends from –*D* to +*D* in the 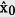 direction [Fig. 1b]. Assuming that the distribution of the height variations *ζ* can be represented by a Gaussian probability function with <ζ> = 0 (i.e. centred at *z* = 0), we have


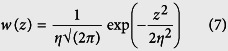


where *η* is the surface roughness. For a complete description of the surface, the knowledge about the density of the height variations on the surface (i.e. the separation between the valleys and crests) is critically important. This information is given by the autocorrelation function


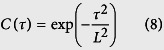


where *τ* = *x*_1_ − *x*_2_ is the separation distance between two repeated height variation features and *L* is the correlation length. An analogy to understand this description is to compare with a periodic surface, where *L* and *η* would correspond to the period and amplitude, respectively. A large *L* means that the surface has correlation at large distances, and this corresponds to gradual slopes for the irregularities.

To obtain the mean value of the scattered field given by Eq. (6), we write


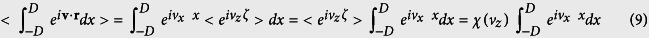


where *χ*(*v*_*z*_) is the characteristic function of a statistical distribution


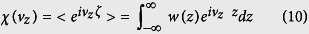


Replacing Eqs (9 and 10) in Eq. (6) we have


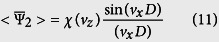


Note that the term sin(*v*_*x*_*D*)·(*v*_*x*_*D*)^−1^ is equal to 1 in the specular direction (i.e. when θ = φ) and rapidly goes to 0 in all other directions. Also note that by definition, the characteristic function for a normal distribution function is 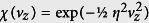.

For our scope of heat transport in semiconductors, the quantity of interest is the energy carried by the scattered wave, which is proportional to the square of the scattered field


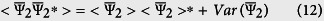


By using Eqs (6) and (9), we can write


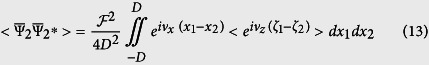


By using statistical definitions of these quantities we can get an expression depending on relevant physical parameters,


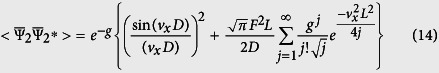


where 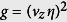. Equation (14) is often referred to as the Beckmann-Kirchhoff surface scattering model. It represents a complex angular distribution of the scattered energy [Fig. 1a] where *the proportion of specularly scattered phonons* from the surface is given by


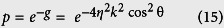


### Shadowing

The function to quantify the shadowing for an arbitrary point on the surface *S*(*θ, x*) is defined as unity for points not in shadow and zero for shaded parts of the surface. The spatial averaging of this function yields the shadowing function. Considering an arbitrary point on the surface at τ = 0, and define the probability,


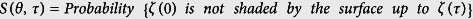


Then *S*(*θ*) is defined as:


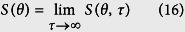


Using appropriate boundary conditions, the shadowing function *S*(*θ*) can be expressed as^52^,


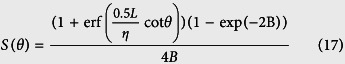



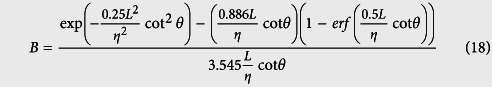


For application to heat transport and models based on the specularity parameter (*p*), we consider a case of bistatic scattering where the scattered angle *ϕ* lies between 0 and *π*/2 radians. In particular, for specular reflection the scattered angle is equal to the incident angle *θ*. The shadowing function thus obtained would accurately account for the surface shadows for the Beckmann-Kirchhoff model previously described, particularly at grazing incidence angles. In particular, the bistatic shadowing function *S*(*θ, ϕ*) in the specular direction (*ϕ* = *θ*) can be expressed as:


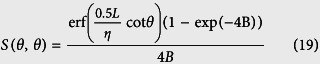


### Mean-Free-Path Reduction

To calculate the reduced mean free paths *ℓ* we use the fundamental definition of the phonon mean free path and consider phonons with wavevector **k** originating at an arbitrary point O within the semiconductor nanowire (see Supplementary Fig. S4). Within the wire, phonons are subject to continuous internal (e.g. Umklapp and impurity) scattering processes, which determine the length of the bulk phonon mean free paths *ℓ*_0_. After continuous internal scattering and successive boundary scattering, the *reduced mean free paths ℓ* for phonons originating at point O are obtained by statistically summing the distances travelled between each successive scattering and are given by:


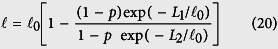


Equation (20) replaces the commonly-used Matthiessen’s rule by correctly considering phonon boundary scattering as a surface effect. Thus, the loss of coherence at the boundaries is incorporated using the Beckmann-Kirchhoff scattering theory, and within the nanowire by considering the relaxation times due to phonon-phonon scattering in the Boltzmann Transport Equation.

## Additional Information

**How to cite this article**: Malhotra, A. and Maldovan, M. Impact of Phonon Surface Scattering on Thermal Energy Distribution of Si and SiGe Nanowires. *Sci. Rep.* **6**, 25818; doi: 10.1038/srep25818 (2016).

## Supplementary Material

Supplementary Information
