# Supplementary material for: Impact of Phonon Surface Scattering on Thermal Energy Distribution of Si and SiGe Nanowires
Source: Sci Rep. 2016 May 13;6:25818. doi: 10.1038/srep25818 (PMC4865844; doi:10.1038/srep25818)
Supplement: Supplementary Information [file srep25818-s1.pdf]

Supplementary Information:

Impact of Phonon Surface Scattering on Thermal Energy Distribution of Si and SiGe Nanowires

Abhinav Malhotra[1], Martin Maldovan[1, 2 *]

[1] *School of Chemical & Biomolecular Engineering, Georgia Institute of Technology, Atlanta, Georgia 30332, USA*

[2] *School of Physics, Georgia Institute of Technology, Atlanta, Georgia 30332, USA*

Applicability Range

A practical question is the considerations that need to be taken when applying Beckmann-Kirchhoff (BK) surface models to nanoscale heat. This is due to the fundamental nature of heat. In electromagnetics and acoustics there exists a possibility for the study to be constrained to a particular wavelength. In contrast, the complete wavelength range of heat-transporting phonons needs to be accounted for in order to generate useful results, making it impossible to conduct such a single wavelength study in heat transport. The BK approach relies on the tangent plane approximation [1] which requires that a tangent to be drawn through a point on the surface. This approximation holds true when the radius of curvature of the surface roughness features is large as compared to the wavelength, but is expected to break down if the irregularities have sharp edges or points. The surface correlation length is an indicator of the existence of these features as a large correlation length indicates a gradually wavering surface, and the tangent plane approximation holds when correlation length is larger than the incident wavelength.

Recent experimental measurements[2-7] indicate that the correlation lengths (L) in nanowires are in the range of 3-12 times roughness (η). Based on these, we performed an analysis of the applicability of BK method in contrast to the Rayleigh Rice (RR) Perturbation Method[8,9] which is also commonly used for surface scattering.

Article	Diameter (nm)	η (nm)	L (nm)	Measured κ (W/m-K)
Li et al. (2003)	22-115	-	-	~1-48
Hochbaum et al. (2008)	50-115	-	-	~1-10
Hippalgaonkar et al. (2010)	79-83	1.97, 7.40	62, >120	~1-25
Lim et al. (2012)	50-99	1.8-4.3	5-15	~1-20
Feser et al. (2012)	110-150	0-4	15-40	~1-40
Ghossoub et al.(2013)	68-123	1-1.5	4-8	~1-15

Supplementary Table S1: Silicon nanowire thermal conductivity measurements.

The investigation of the applicability regime for the BK and RR methods in the context of heat transport is the first of its kind. We proceed by focusing on the specularity parameter p and its dependence on surface features. The plot of the specularity parameter obtained from the RR approach, and the BK method for normal incidence and representative incident angles of 30°, 50° and 70° is shown in Figure S2. It may be noted that normal incidence (the Ziman formula) is a particular case within the general BK approach. For the purpose of this study, we consider surface roughnesses η=0.1 and 0.9 nm. It needs to be mentioned that a roughness of 0.1 nm corresponds to variations far less that of a silicon unit cell and can be considered practically a smooth surface. We also note that, from an experimental point of view, the majority of purposely roughened nanowires reported in literature have surface correlation lengths in the range of 4-10 times the roughness (see Table 1). We carry out our analysis in the Brillouin space and the specularity parameter is plotted for two example correlation values (L=5η, 10η) for each of the above roughnesses using both models. It is seen that for very smooth interfaces (η=0.1nm), the perturbation approach has a higher domain of applicability in the k-space and is shortened linearly with increasing roughness. At low roughness and low correlation lengths, the tangent plane approximation does not hold within the silicon k-space. For higher correlation lengths, it is easily visualized that the BK model can be applied. The applicability regime of BK method is increased with higher correlation lengths which is expected as more wave-vectors lie in the

region where the tangent plane approximation holds. Furthermore, it can be observed that the BK approach has higher applicability for increasing roughness values, while the RR method becomes less applicable. Broadly speaking, the applicability of BK method is for higher values of k-vectors and valid across a large k-space. The RR approach is better suited for lower values of k-vectors. Note that the Ziman formula $p = \exp(-4\eta^2 k^2)$ overestimates the scattering and basically leads to completely diffuse scattering for its applicability range.

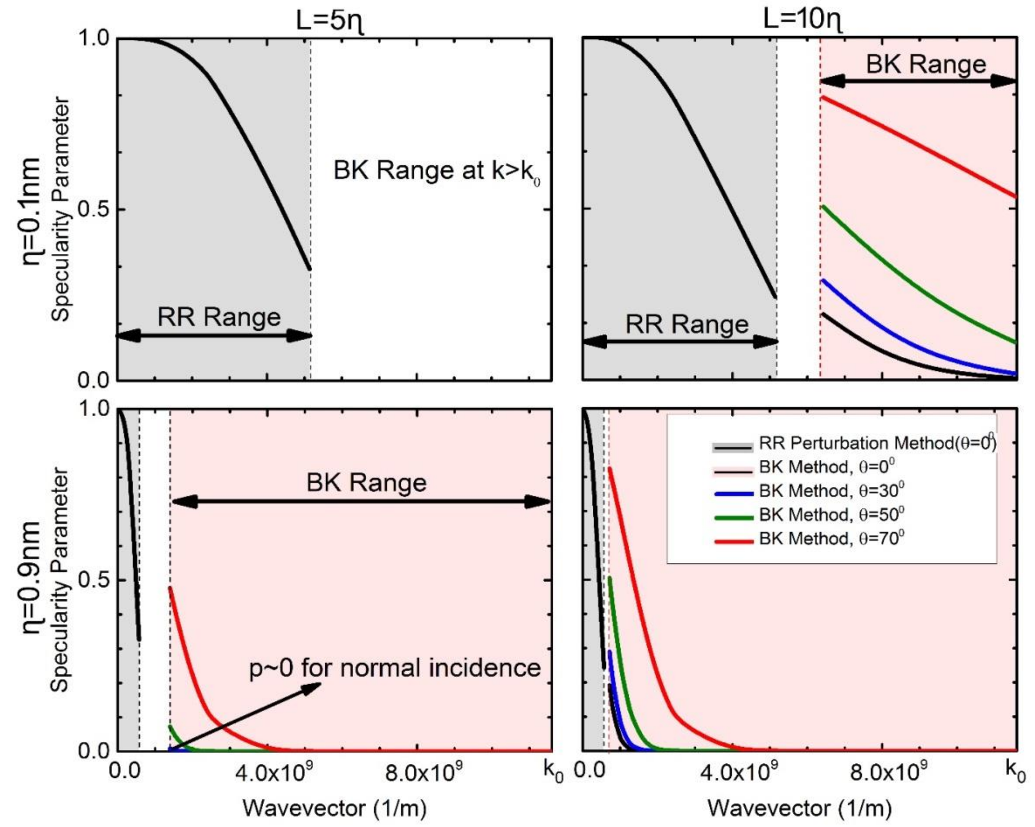

Supplementary Figure S2: Applicabiltiy ranges for Rayleigh-Rice (RR) perturbation approach and Beckmann-Kirchhoff (BK) model (for different angles of incidence) and their applicability range across silicon wavevector k-space.

Bulk Silicon Relaxation Times

The phonon relaxation times for bulk silicon at room temperature from various approaches are plotted in Figure S3. Overall, the different approaches[10-14] yield the same range of relaxation times. The results show that models obtained by different approaches vary at very low frequencies, however it is important to note that low frequency phonons are non-dominant heat carriers in Si at room temperatures. The close agreement between the results from the approach used in this study[11,15] and DFT calculations[12,13] especially for the transverse branch (dominant for heat transport) is noteworthy.

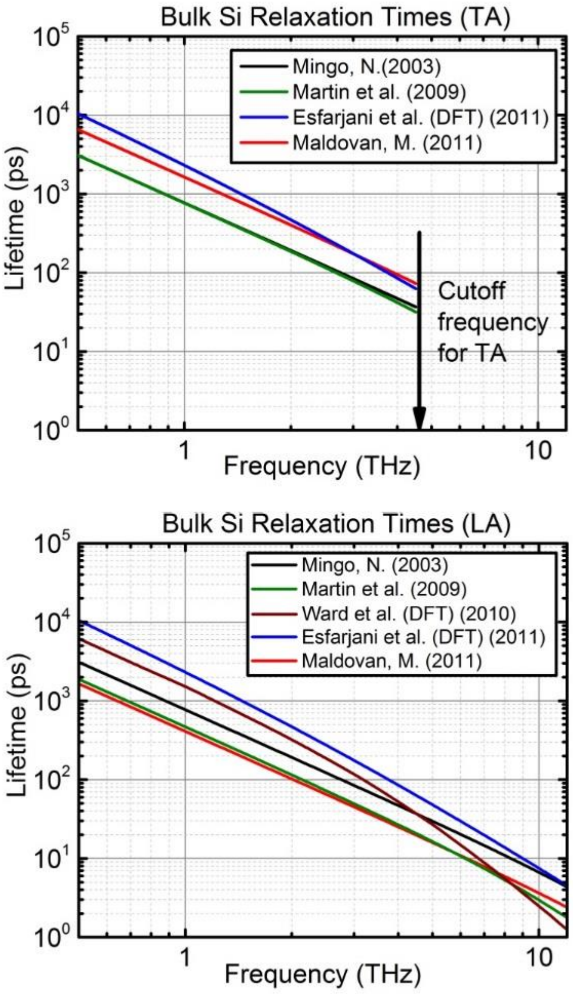

Supplementary Figure S3: Comparison of relaxation times in bulk Si from literature.

Reduced Phonon Mean Free Paths

The proportions of specularly and diffusely scattered phonons at the surface are given by p and $1-p$ respectively. Since diffuse scattering randomizes momentum, a proportion $1-p$ of phonons arriving at the surface does not contribute further to heat transport. To calculate ℓ, we need to consider that phonons, in general, can strike the nanowire surfaces several times. The distance traveled by phonons between these events is constant and given by $L_2=X'X''$. Note that the probability density function for phonon-phonon scattering as phonons propagate internally along the direction ξ is given by

$$dP = \frac{1}{\ell_0} \exp\left(-\frac{\xi}{\ell_0}\right) d\xi \tag{1}$$

By using the fundamental definition of the phonon mean-free-path [16]

$$\ell = \int_0^\infty \xi \, dP \tag{2}$$

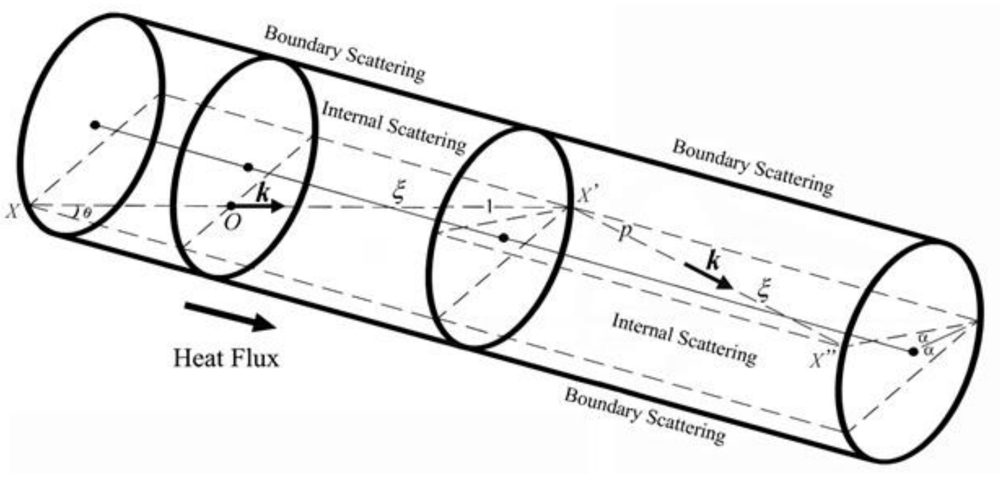

Supplementary Figure S4: Schematic showing the succesive boundary scattering for phonons with wavevector **k** originating at *O*.

the reduced mean-free-paths ℓ for phonons originating at point O after successive specular and diffuse surface scattering (in addition to phonon-phonon internal scattering) is given by

$$\begin{aligned}\ell = &\int_0^{L_1} \xi \frac{1}{\ell_0} \exp\left(-\frac{\xi}{\ell_0}\right) d\xi + (1-p)\int_{L_1}^{\infty} L_1 \frac{1}{\ell_0} \exp\left(-\frac{\xi}{\ell_0}\right) d\xi + \\ &p\int_{L_1}^{L_1+L_2} \xi \frac{1}{\ell_0} \exp\left(-\frac{\xi}{\ell_0}\right) d\xi + p(1-p)\int_{L_1+L_2}^{\infty} (L_1+L_2) \frac{1}{\ell_0} \exp\left(-\frac{\xi}{\ell_0}\right) d\xi + \ldots\end{aligned} \quad (3)$$

where the first term is the contribution from phonons that were scattered by other phonons before reaching the surface at point X' (i.e. along $L_1 = OX'$), the second term is the contribution from phonons that would have traveled beyond X' but were diffusely scattered at X', the third term is the contribution from phonons that traveled from X' to X'', and so forth. The above series converges to the function

$$\ell = \ell_0 \left[1 - \frac{[1-p]\exp[-L_1/\ell_0]}{1-p\exp[-L_2/\ell_0]} \right] \quad (4)$$

which gives the reduced phonon mean-free-paths ℓ in the nanowire. We note that in Eq. (4), $\ell=\ell(\mathbf{k})$, $p=p(\mathbf{k})$, $L_1=L_1(\mathbf{k})$, and $L_2=L_2(\mathbf{k})$ are all dependent on the wavevector **k** (or equivalently frequency ω).

Thermal Conductivity

If a time-independent spatially uniform thermal gradient $\partial T/\partial x$ is applied along the axial x direction of the nanowire, by definition, the heat flux along x can be calculated as

$$J_x = \frac{1}{(2\pi)^3} \int \hbar\omega_{\mathbf{k}} \; f_{\mathbf{k}} \; v_{x,\mathbf{k}} \; d\mathbf{k} \quad (5)$$

where $\hbar$ is the Planck constant, $\omega_{\mathbf{k}}$ is the phonon frequency, $f_{\mathbf{k}}$ is the distribution function, $v_{x,\mathbf{k}}$ is the velocity along x, and the integral is performed over all wavevectors **k** (including transverse and longitudinal phonon polarizations).

In addition, the solution of the linear Boltzmann transport equation (under the relaxation time approximation) where $f_{\mathbf{k}}$ is expanded as $f_{\mathbf{k}} = f_{\mathbf{k}}^0 + f_{\mathbf{k}}^1$ with $f_{\mathbf{k}}^1 << f_{\mathbf{k}}^0$, establishes that[17]

$$f_{\mathbf{k}}^1 = -\tau_{\mathbf{k}} v_{x,\mathbf{k}} \frac{\partial f_{\mathbf{k}}^0}{\partial T} \frac{\partial T}{\partial x} \tag{6}$$

Combining Eqs. (5) and (6), the nanowire thermal conductivity κ, where $J_x = -\kappa\,(\partial T/\partial x)$, is

$$\kappa = \frac{1}{(2\pi)^3} \int \hbar\omega_{\mathbf{k}} \, (\tau_{\mathbf{k}} v_{x,\mathbf{k}} \frac{\partial f_{\mathbf{k}}^0}{\partial T}) \, v_{x,\mathbf{k}} \, d\mathbf{k} \tag{7}$$

where $f_{\mathbf{k}}^0 = [\exp(\hbar\omega_{\mathbf{k}} / k_B T) - 1]^{-1}$ is the Bose-Einstein equilibrium distribution function. Note that Eq.(7) is the local thermal conductivity $\kappa=\kappa(\mathbf{r})$ since the expression depends on the location inside the nanowire due to the dependence of the *reduced* mean free path $\ell_{\mathbf{k}}=\tau_{\mathbf{k}} v_{\mathbf{k}}$ on $\mathbf{r}$. The overall nanowire thermal conductivity is calculated by integrating the local thermal conductivity over the cross section of the nanowire. We note that the above formulation for phonon transport is equivalent to analytically solving the Boltzmann transport equation inside the nanowire, as done by Sondheimer[18]. However our approach preserves the explicit expression for the reduced phonon mean free paths.

Comparison of Phonon Boundary Scattering

A comparison between the heat spectrum calculations from our model which includes all physical parameters, and a model that assumes normal incidence for all phonons (i.e. Ziman formula) is shown in Figure S5. For a nanowire of d=100nm and a small roughness of 0.3nm, Ziman formula overpredicts the scattering to be nearly diffusive (dotted line). The normal incidence assumption over-predicts the heat carried by shorter wavelengths and causes a prediction of a more diffusively scattering boundary than what occurs physically.

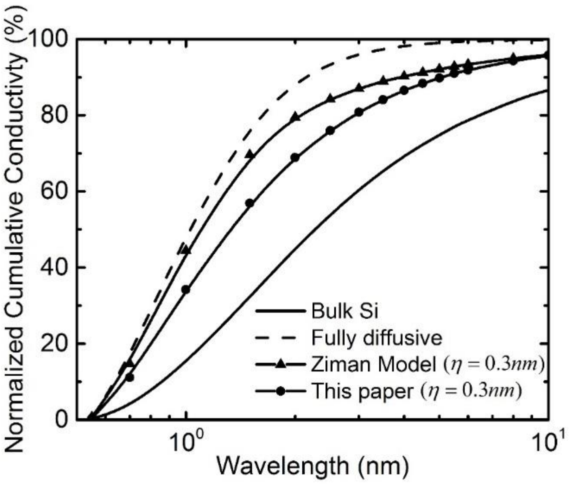

Supplementary Figure S5: Calculated wavelength heat spectrum for a nanowire with diameter d=100nm using our physical approach (BK model with shadowing) and simplified approach (Ziman) which ignores the dependence on incident angle. Bulk silicon and frequency-independent fully diffusive boundary scattering are shown as reference for the limiting cases.

Confinement Contribution for Si and SiGe Nanowire with Roughness *0.5nm*

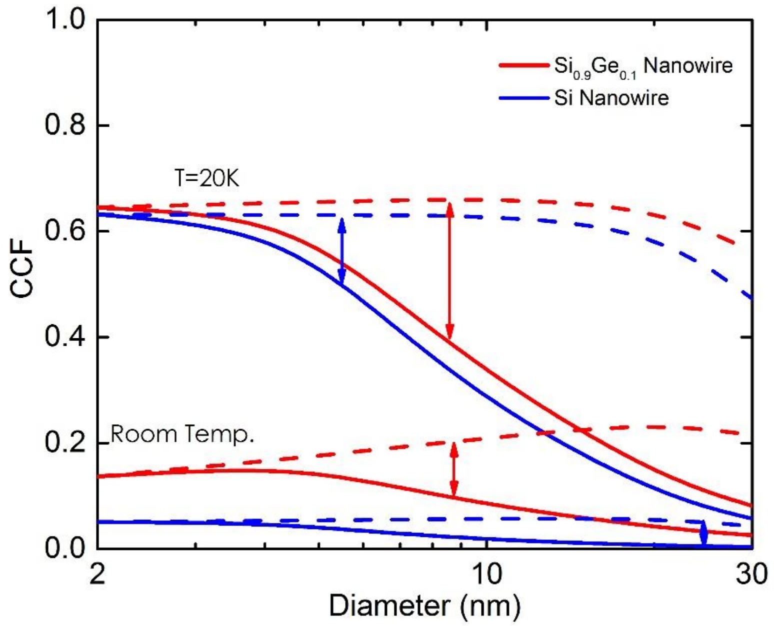

Supplementary Figure S6: CCF values (minimum in solid and upper range as dashed line) for Si and SiGe nanowires for a roughness of 0.5nm and correlation length $L=10\eta$.

References

1 Beckmann, P. & Spizzichino, A. *The scattering of electromagnetic waves from rough surfaces*. Vol. 4 (Pergamon Press Limited, 1987).

2 Ghossoub, M. G. *et al.* Spectral phonon scattering from sub-10 nm surface roughness wavelengths in metal-assisted chemically etched Si nanowires. *Nano Lett.* **13**, 1564-1571, (2013).

3 Lim, J., Hippalgaonkar, K., Andrews, S. C., Majumdar, A. & Yang, P. Quantifying surface roughness effects on phonon transport in silicon nanowires. *Nano Lett.* **12**, 2475-2482, (2012).

4 Feser, J. P. *et al.* Thermal conductivity of silicon nanowire arrays with controlled roughness. *J. Appl. Phys.* **112**, 114306, (2012).

5 Hippalgaonkar, K. *et al.* Fabrication of microdevices with integrated nanowires for investigating low-dimensional phonon transport. *Nano Lett.* **10**, 4341-4348, (2010).

6 Hochbaum, A. I. *et al.* Enhanced thermoelectric performance of rough silicon nanowires. *Nature* **451**, 163-167, (2008).

7 Li, D. *et al.* Thermal conductivity of individual silicon nanowires. *Appl. Phys. Lett.* **83**, 2934, (2003).

8 Bass, F. G. & Fuks, I. M. *Wave Scattering from Statistically Rough Surfaces*. (Pergamon Press, 1979).

9 Voronovich, A. G. *Wave Scattering from Rough Surfaces*. (Springer, 1999).

10 Mingo, N. Calculation of Si nanowire thermal conductivity using complete phonon dispersion relations. *Phys. Rev. B* **68**, (2003).

11 Maldovan, M. Micro to nano scale thermal energy conduction in semiconductor thin films. *J. Appl. Phys.* **110**, 034308, (2011).

12 Esfarjani, K., Chen, G. & Stokes, H. T. Heat transport in silicon from first-principles calculations. *Phys. Rev. B* **84**, (2011).

13 Ward, A. & Broido, D. A. Intrinsic phonon relaxation times from first-principles studies of the thermal conductivities of Si and Ge. *Phys. Rev. B* **81**, (2010).

14 Martin, P., Aksamija, Z., Pop, E. & Ravaioli, U. Impact of phonon-surface roughness scattering on thermal conductivity of thin si nanowires. *Phys. Rev. Lett.* **102**, 125503, (2009).

15 Maldovan, M. Thermal conductivity of semiconductor nanowires from micro to nano length scales. *J. Appl. Phys.* **111**, 024311, (2012).

16 Reif, F. *Fundamentals of Statistical and Thermal Physics*. (McGraw-Hill, 1965).

17 Ziman, J. M. *Electrons and Phonons*. (Oxford University Press, 1960).

18 Sondheimer, E. H. The mean free path of electrons in metals. *Adv. Phys.* **1**, 1, (1952).
